# Exploiting combinatorial cultivation conditions to infer transcriptional regulation

**DOI:** 10.1186/1471-2164-8-25

**Published:** 2007-01-22

**Authors:** Theo A Knijnenburg, Johannes H de Winde, Jean-Marc Daran, Pascale Daran-Lapujade, Jack T Pronk, Marcel JT Reinders, Lodewyk FA Wessels

**Affiliations:** 1 Information and Communication Theory Group, Faculty of Electrical Engineering, Mathematics and Computer Science, Delft University of Technology, Mekelweg 4, 2628 CD Delft, The Netherlands; 2 Industrial Microbiology, Department of Biotechnology, Delft University of Technology, Julianalaan 67, 2628 BC Delft, The Netherlands; 3 Department of Molecular Biology, The Netherlands Cancer Institute, Plesmanlaan 121, 1066 CX Amsterdam, The Netherlands

## Abstract

**Background:**

Regulatory networks often employ the model that attributes changes in gene expression levels, as observed across different cellular conditions, to changes in the activity of transcription factors (TFs). Although the actual conditions that trigger a change in TF activity should form an integral part of the generated regulatory network, they are usually lacking. This is due to the fact that the large heterogeneity in the employed conditions and the continuous changes in environmental parameters in the often used shake-flask cultures, prevent the unambiguous modeling of the cultivation conditions within the computational framework.

**Results:**

We designed an experimental setup that allows us to explicitly model the cultivation conditions and use these to infer the activity of TFs. The yeast *Saccharomyces cerevisiae *was cultivated under four different nutrient limitations in both aerobic and anaerobic chemostat cultures. In the chemostats, environmental and growth parameters are accurately controlled. Consequently, the measured transcriptional response can be directly correlated with changes in the limited nutrient or oxygen concentration. We devised a tailor-made computational approach that exploits the systematic setup of the cultivation conditions in order to identify the individual and combined effects of nutrient limitations and oxygen availability on expression behavior and TF activity.

**Conclusion:**

Incorporating the actual growth conditions when inferring regulatory relationships provides detailed insight in the functionality of the TFs that are triggered by changes in the employed cultivation conditions. For example, our results confirm the established role of TF Hap4 in both aerobic regulation and glucose derepression. Among the numerous inferred condition-specific regulatory associations between gene sets and TFs, also many novel putative regulatory mechanisms, such as the possible role of Tye7 in sulfur metabolism, were identified.

## Background

The simple and often used biological model to unravel transcriptional regulation ascribes the change in gene expression levels, as observed between different cellular conditions, to changes in the activity of transcription factors (TFs). Change of the transcriptional activity of a TF is one of the means by which an organism adapts to changes in the extracellular environment. A substantial amount of research has employed this model to infer regulatory networks by integrating gene expression data, sequence data (to detect the *cis*-regulatory binding sites of TFs), e.g. [1-3], and/or TF binding data, e.g. [4-6]. For an overview see [7-9]. In most cases, the generated regulatory networks are derived from large microarray compendia. Notwithstanding the many advantages of such approaches, two main drawbacks can be identified. Firstly, these compendia gather very heterogeneous gene expression data derived from various culture conditions (media, pH, temperature, etc.) that, in a large majority of the cases, solely compare the culture conditions to their direct condition-specific references. Different cultivation conditions within the compendium can, therefore, hardly be compared. Secondly, the interpretation of transcriptome data obtained from the generally employed shake-flask cultivations is likely to be complicated by differences in specific growth rate, carbon catabolite repression, nitrogen catabolite repression, and more generally continuous changes in environmental conditions. This prevents the establishment of a direct link between the activity of TFs and specific growth conditions.

A frequently employed approach links a TF to a module, i.e. a set of co-expressed genes, based on TF binding data or promoter analysis. Enrichment of functional categories (such as GO [10] and MIPS [11]) within the module provides clues about the function of the TFs associated with the module. Although this can provide a global view of the transcriptional role of a TF, we are convinced that the precise conditions or perturbations that trigger a change in the activity of TFs should be an integral part of the generated regulatory network.

**Figure 1:**
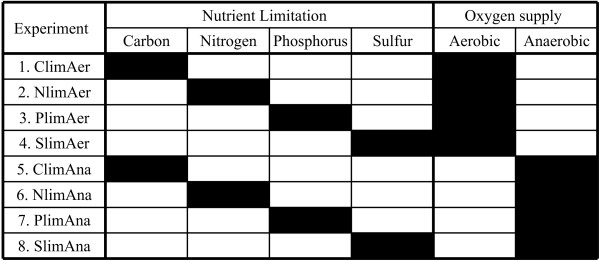
**Schematic overview of the combinatorial cultivation conditions**. Black squares indicate the employed nutrient limitation and oxygen supply.

To this end, we designed an experimental setup that allowed us to explicitly model the cultivation conditions and use these to infer the activity of TFs. To achieve this, we employed chemostat cultures that enable the cultivation of micro-organisms under tightly defined environmental conditions. Chemostat cultures are superior to the shake-flask cultures in both accuracy and reproducibility [12]. In a chemostat, culture broth (including biomass) is continuously replaced by fresh medium at a fixed and accurately determined dilution rate. When the dilution rate is lower than *μ*_*max*_, the maximal specific growth rate of the micro-organism, a steady-state situation will be established in which the specific growth rate equals the dilution rate. In such a steady-state chemostat culture, *μ*, is controlled by the (low) residual concentration of a single growth-limiting nutrient. In this research, microarrays were employed to measure the genome-wide transcriptional response of the yeast *Saccharomyces cerevisiae *to growth limitation by four different macronutrients (carbon, nitrogen, phosphorus, and sulfur) in both aerobic and anaerobic chemostat cultures (Figure 1) [13]. Except for the different nutrient limitations and oxygen availability, all other culture parameters (such as growth rate, pH, temperature, etc.) were kept constant throughout the different experiments. Thus, changes in gene expression levels can solely be attributed to the different nutrient limitations and the oxygen regime. We devised a computational approach that exploits the interrelatedness between the conditions in order to identify the individual and combined effects of nutrient limitations and oxygen availability on expression behavior and TF activity. The inclusion of the growth conditions in the analysis allows for the identification of direct links between the cultivation conditions, TFs triggered by specific cultivation conditions and the targets of these TFs.

## Results

### Overview of the computation approach

**Figure 2:**
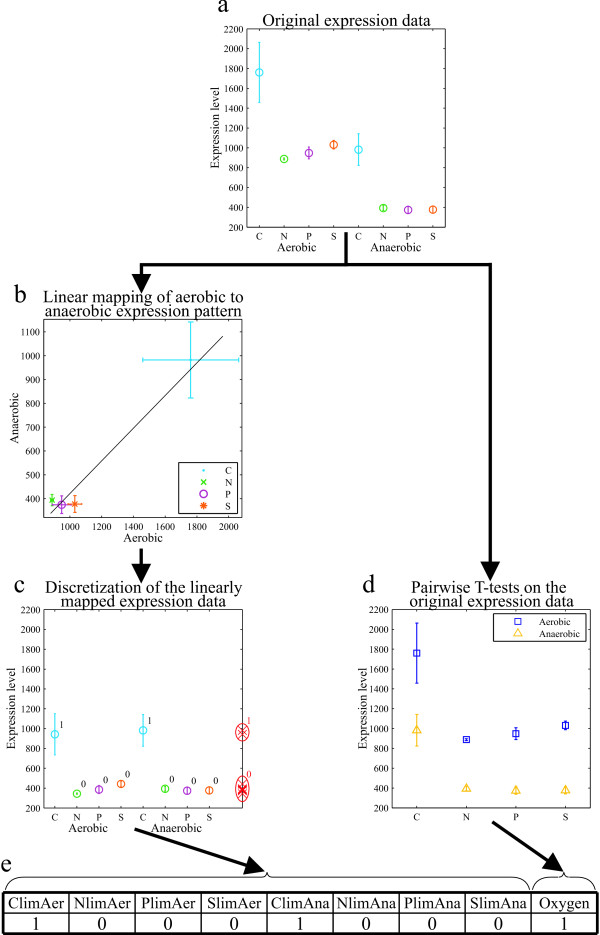
**Procedure to derive the discretized representation of a gene**. **a) **Examination of the expression levels under the eight cultivation conditions led to the observation that for many genes the expression pattern across the four nutrient limitations when grown aerobically is a scaled and offset version of its anaerobic counterpart. (Permutation tests were performed to confirm this notion (Additional file 3)). **b) **This "global oxygen effect", i.e. the effect that presence of oxygen has on the expression levels across all or most of the nutrient limitations, is modeled as a linear relationship and estimated using a regression strategy. **c) **The estimated regression parameters (slope and offset) are employed to isolate the oxygen effect by transforming the aerobic expression values. Discretization of this pattern allows for identification of up- or downregulation as a consequence of specific nutrient limitations and possible nutrient-limitation-specific effects of oxygen presence. **d) **Pairwise T-tests are performed to compare the original aerobic and anaerobic expression values for each nutrient limitation. The results of these tests are combined to detect possible consistent and significant higher or lower expression as a consequence of oxygen presence. **e) **The derived discrete representation of the expression of a gene is visualized in a nine-bit ternary (-1,0,1) vector.

**Figure 3:**
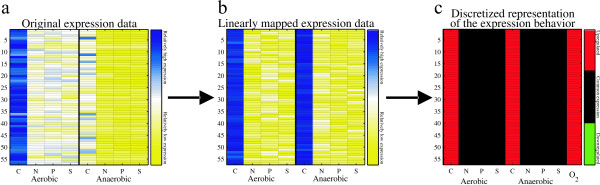
**Heatmaps of a module**. **a) **Normalized expression pattern of all (57) genes that share the same discretized representation, namely 100010001, and consequently, form a module. This representation, which indicates upregulation under carbon limitation and higher expression when grown within the presence of oxygen, is identical to the one derived in Figure 2. The expression patterns of the genes in this heatmap are comparable to the expression pattern in Figure 2a. **b) **Normalized expression pattern of the genes after the linear mapping is applied. Isolation of the oxygen effect clearly reveals upregulation under the carbon limitation. The linearly mapped expression patterns are comparable to the one in Figure 2c. **c) **The (identical) discretized expression pattern for the 57 genes. Note that our discretization procedure assigns a 0 to the cultivation conditions that form the most common expression level. For these 57 genes this common expression level is represented in **b **by the dark yellow, which occurs in six of the eight conditions. The ninth entry of this representation, i.e. the oxygen effect, is also characterized as upregulated, since the original expression levels in **a **are consistently higher under aerobic growth when compared to anaerobic growth.

From the continuous expression levels measured across the cultivation conditions we derive a discretized representation of the expression behavior for each gene. This representation indicates up- or downregulation as a consequence of the individual or combined effects of the nutrient limitations and oxygen availability. Here, we exploit the combinatorial setup of the cultivation conditions to recognize and dissect the effect of the presence of oxygen on the expression levels of a gene. More specifically, we employ a regression strategy to detect, model and correct for the effect of oxygen presence. This procedure is outlined in Figure 2 and explained in detail in the Methods section. Modules are generated by clustering genes with identical expression representations (Figure 3). Next, we integrate TF binding data [14] to assess whether a TF or a pair of TFs binds the promoter regions of a module much more frequently than would be expected by chance. A significant relationship between a module and a TF suggests that the TF is (partly) responsible for the expression behavior of that particular module. Since the expression behavior of a module reveals under which combination of cultivation conditions the genes are up- or downregulated, we are not only able to relate TFs to the groups of genes that they presumably regulate, but also to the precise environmental conditions that trigger their activity to perform their regulatory role.

### Overview of the uncovered regulatory relationships

**Figure 4:**
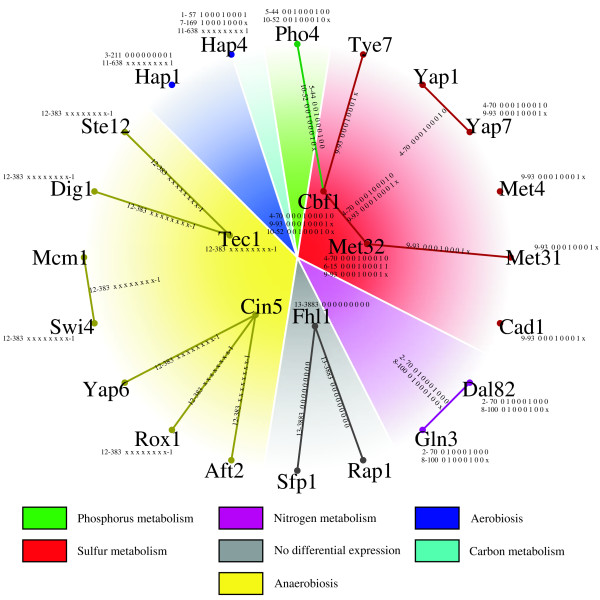
**TF Circle**. The TF circle depicts all the TFs and TF pairs, which are significantly related to at least one module. Related modules are represented by strings in the vicinity of the relevant TF or, in the case of a TF pair, in the vicinity of the line connecting both TFs. The strings are made up out of three parts. The first number represents the number that was assigned to the module. The second number indicates the number of genes in the module. The third part is the discretized expression pattern of the genes in the module. Here, an 'x' indicates the irrelevance (don't care) of a particular cultivation parameter. The color coding of the circle is based on the discretized expression representation of the modules. The placement of the TFs (near the center or the edge) is for reasons of visibility only.

The TF circle (Figure 4) depicts an overview of all the TFs, which are significantly related to one or more modules. In addition, pairs of TFs that can bind the promoter region of the genes in a module significantly often, are connected by a solid line. In the TF circle, the modules and their associated TFs are categorized according to the cultivation parameters under which the genes in the module are differentially regulated, i.e. where the discretized representation differs from zero. This arrangement is given by the color coding of the segments in the circle. From this it is clear which cultivation parameters affect the activity of a TF. Additional information concerning enrichment of gene annotation categories and results of motif discovery in promoter regions of the genes within the modules can be found in Table 1 and more comprehensively in Additional file 1.

In the remainder of this section, modules connected to anaerobiosis, aerobiosis and sulfur metabolism, are discussed in more detail. However, first we consider Module 13 (grey segment in Figure 4) that contains all genes that do not exhibit differential expression between the eight experimental conditions. (The discretized expression pattern consists of all zeros.) Three regulators have been assigned to this module, Fhl1, Sfp1 and Rap1. All three TFs are known to play an essential role in the regulation of ribosomal protein genes [15-17]. Although the strains were grown under different nutrient limitations and oxygen regime, the dilution rate (in other words the growth rate) of *Saccharomyces cerevisiae *was kept equal (0.1 h^-1^) during the chemostat steady state in all the fermentation conditions tested [12,13]). Given that expression regulation of ribosomal protein genes is one of the end targets of the Tor (target of rapamycin) signaling pathway, our results suggest that the regulation through the Tor signalling cascade is independent of the applied nutrient limitation and oxygen availability, but would rather reflect how the cell senses the limiting nutrient to maintain a determined growth rate.

### Controlling anaerobiosis

Module 12 (yellow segment in Figure 4) comprises all (383) genes that show consistent upregulation under anaerobic conditions, irrespective of any nutrient condition. Note that our strategy enables us to isolate the effect that the presence of oxygen has on the expression level of a gene. This offers the obvious advantage to independently analyze this effect. The irrelevance of the nutrient limitations is indicated by 'x's in the discretized representation of Module 12 in Figure 4. Several TFs and TF pairs were found to be able to bind the genes of this anaerobiosis module significantly often. Current knowledge on gene expression regulation under anaerobic conditions cannot explain all the regulatory relationships and related TFs. The anaerobic growth conditions within our systematic experiments can therefore contribute to elucidate the role of several regulators in the absence of oxygen.

The identification of Rox1, already known to play a role in low oxygen processes, objectively validates the truthfulness of this analysis. According to [4], this heme-dependent transcriptional repressor of hypoxic genes [13,18] constitutes a multi-component transcription factor loop together with Yap6 and Cin5, i.e. these three TFs form a regulatory circuit in which they regulate each other. Although our algorithm does not explore these kind of network structures, we identify the concerted regulation amongst these three TFs and based on our results can hypothesize that this loop is active under anaerobic conditions. Additionally, we find the pair Ste12 and Tec1 which is known to activate genes associated with pseudohyphal growth, as well as Dig1, which conversely is involved in the negative regulation of genes involved in pseudohyphal growth [19]. (We observed a large overlap between the genes in the regulon of Tec1-Dig1 and those in the "conjugation with cellular fusion" GO-category (*P *= 6.7·10^-8 ^according to the hypergeometric test)). Finally, the TF pair Mcm1 and Swi4 is connected to anaerobiosis, although both are known to be involved in controlling cell cycle [20]. Moreover, Mcm1 (also named PRTF for "Pheromone Receptor Transcription Factor" [21]) is also involved in mating and response to pheromone, relating it to the cluster of Ste12, Tec1 and Dig1. These results correlate with the observation that *Saccharomyces cerevisiae *grown under anaerobic conditions exhibits elongated cell-shape irrespective of the applied nutrient limitation (See Additional file 6). Further investigation is needed to gain more insight into the role of these regulators in control of anaerobiosis.

Missing from the TFs significantly related to the anaerobiosis module is Upc2, which together with Rox1 is involved in regulating the expression of many genes induced under anaerobic conditions [13,22]. The reason for not retrieving Upc2 is simply the absence of this TF in the genome-wide location analysis employed to build the TF database. Employing motif discovery, however, the aerobic regulator 1 (AR1) binding motif of Upc2 (TCGTT [22]) was found 244 times in the upstream regions of the 383 genes (*P *= 2.4·10^-13^) (See Table 1).

### Controlling aerobiosis

The TFs Hap1 and Hap4 are associated with the regulation of aerobiosis (dark blue segment in Figure 4). Hap1 is solely connected to the presence of oxygen (Modules 3 and 11), while Hap4 is also connected to carbon-limitation (Modules 1 and 7). This is in agreement with a role for Hap4 in both aerobic regulation and glucose derepression [23]. Amongst the targets of Hap1, which are overrepresented in Modules 3 and 11, we find well-known oxygen specific Hap1 regulated genes such as *CTT1, CYB2 *and *CYC1*, confirming that its regulatory role is linked to the presence of oxygen irrespective of limited or high glucose availability. The presence of Hap4 as part of the Hap2/Hap3/Hap4/Hap5 complex fits with the enrichment in energy categories in the aerobic genes (see Table 1 and Additional file 1). This is in line with the involvement of the Hap complex in the regulation of mitochondrial functions such as TCA cycle, electron transport chain and respiration. However, overrepresentation of only Hap4 targets from the location analysis dataset may appear as a surprise. Overrepresentation of Hap2 or Hap3 may be expected, because these two subunits of Hap2/Hap3/Hap4/Hap5 actually bind the DNA, while Hap4, as a regulatory subunit, does not.

Furthermore, a clear-cut discrepancy exists between the location analysis data of the separate members of the Hap complex. The results of this study imply that the TF binding data of Hap4 is the more relevant one. This would then suggest that in order to monitor the DNA binding of a transcriptional complex, e.g. Hap2/Hap3/Hap4/Hap5, it would be more suitable to tag the subunits that do not bind the DNA template, speculating that tagging DNA binding subunits may alter the structure of the complex and, consequently, the affinity and the specificity of the interaction with the DNA.

### Sulfur metabolism

**Figure 5:**
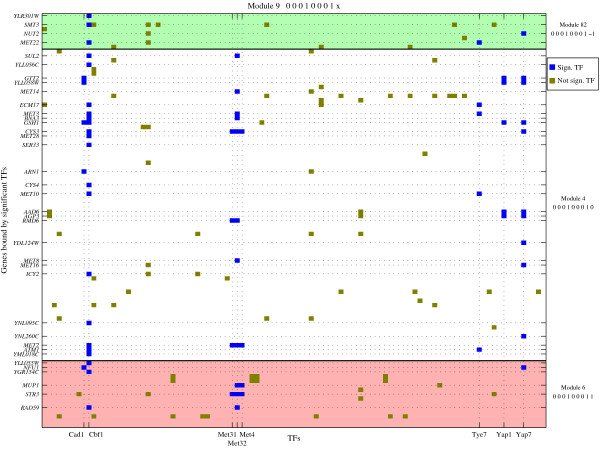
**TF-Gene Map for Module 9**. The TF-Gene map indicates which genes in the module can be bound (upstream) by the TFs that are significantly related to this module. Only those genes that have a binding motif in their upstream region for one of these significant TFs are annotated along the vertical axis. For these genes a dotted horizontal line is drawn. The significant TFs are annotated along the horizontal axis. For these TFs a dotted vertical line is drawn. This module, which contains all genes upregulated under sulfur limitation irrespective of the oxygen effect, can be subdivided into groups characterized by their response to oxygen presence. Genes at the top of the map (with green background) have a significantly lower expression when grown without the presence of oxygen. This group corresponds to Module 82. The middle part of the map (with white background) displays genes, which are not affected by the presence of oxygen. This group corresponds to Module 4. Genes in the bottom of the map (with red background) have higher expression when grown aerobically. This group corresponds to Module 6.

The systematic combinatorial setup of cultivation conditions used to generate the transcript data allows us to extract specific information on genes regulated in response to a certain nutrient limitation. Modules 9, 6 and 4 and 82 form prime examples. Module 9 (red segment of the circle) contains all (93) sulfur-limitation-upregulated genes, regardless of the effect that the presence of oxygen might have on the expression of the genes. Modules 6, 82 and 4 consist of the sulfur-limitation-upregulated genes for which oxygen presence leads to higher expression (15 genes), lower expression (8 genes, not in Figure 4) and no significant change in expression (70 genes). Thus, Module 9 is the union of Modules 6, 4 and 82. Figure 5 displays genes from Module 9 that are bound by the TFs, which are significantly related to the set of sulfur regulated genes. In this map, genes are subdivided into groups based on their response to oxygen presence. Several genes that show either a higher or lower expression level depending on oxygen presence, i.e. genes from Module 6 and 82 respectively, also have a binding site for the significant TFs. For example, *MET22*, involved in methionine biosynthesis, exhibits higher expression when grown anaerobically. This can be related to the fact that the promoter sequence of *MET22 *contains a LORE (low oxygen response element) motif [24], which provides clues for future research to elucidate the functionality of this gene. Amongst the genes that have a higher expression when grown aerobically and that are bound by significant TFs, is *STR3*, involved in homocysteine and cysteine interconversion that is part of the sulfur amino acid biosynthesis and sulfur degradation pathway. Currently no relationship is known between sulfur- and oxygen-dependent regulation of this gene.

The regulatory network constructed from our analysis reveals a complex interplay between six individual transcription factors (Met4, Met31, Met32, Cbf1, Yap7 and Cad1) and four pairs of regulators (Tye7-Cbf1, Cbf1-Met32, Met32-Met31 and Yap1-Yap7) connected to sulfur metabolism. Met4, Met31, Met32 and Cbf1 constitute an internal validation of the analysis, since these four factors are indeed known as members of the Met regulatory complex [25] that also includes the regulatory subunit Met28. More interestingly, our data provide new insight into sulfur metabolism regulation by implicating new regulators as Tye7 and the members of the fungal-specific family of basic leucine zipper (bZIP) proteins Yap1, Cad1 (Yap2) and Yap7. Literature reports available so far concerning Tye7 limit its role to cell cycle [26]. Our results, however, would implicate that Tye7 in combination with Cbf1 would participate in the regulation of the genes encoding the upper part of the sulfur assimilation pathway including *MET3*, *MET10*, *ECM17*, *MET22* and *ATM1*, who's gene products are involved in maturation of cytosolic Fe/S (iron-sulfur) proteins [27]. Even more interesting is the possible cross-coupling with phosphate metabolism. As indicated in Figure 4, Cbf1 was also found to bind the upstream regions of phosphorus regulated genes significantly often. Given that Cbf1, Pho4 and Tye7 recognize similar binding sites, our results could shed new light on the possible cross-regulation of phosphate and sulfate metabolism that centers around Pho4 and Cbf1 [28].

In the case of Cad1 and Yap1 the link to sulfur metabolism may correlate to their reported role in mediating resistance to cadmium (Cd^2+^), which leads to changes in the sulfate assimilation pathway and to sulfur sparing [29]. When *Saccharomyces cerevisiae *is exposed to Cd^2+ ^most of the sulfur assimilated by the cells is converted into glutathione, a thiol-metabolite essential for detoxification. Yeast adapts to this vital metabolite requirement by globally modifying its proteome to reduce the production of abundant sulfur-rich proteins.

## Discussions and conclusion

We observed and successfully modeled that the presence of oxygen leads to an offset (addition) and/or scaling (multiplication) of the expression levels of many genes, corroborating the existence of various types of regulation on various levels. The uncovered results find their origin in the systematic combinatorial setup of the well-defined cultivation conditions within the experiment. Our tailored approach exploits the interrelatedness between the conditions and links the cultivation parameters to TF activity and gene expression behavior.

We compared our strategy to an approach that follows the exact same steps, but which does *not *exploit the systematic setup of the cultivation conditions. In short, when the interrelatedness between the conditions is not used, the original continuous expression levels are discretized without modeling the oxygen effect. Results of this comparison indicate that more genes can be related to a particular cultivation parameter when incorporating the relations between the cultivation conditions. See Table 2. Additionally, we can relate more TFs and TF pairs to the generated modules and achieve higher functional annotation enrichment. See Additional files 4 and 5 (as well as Additional files 1 and 2 for a more in depth comparison). These results provide additional evidence for the validity of the adopted approach. Moreover, the inclusion of the conditions within the computational framework accommodates the assessment of the direct effect of these conditions on gene expression, TF activity and other biological processes in the cell. This is in contrast to the currently used compendium approaches, where the relation between the cultivation conditions is ambiguous and can not be modeled. There, large heterogeneity in terms of the strain, growth rate, growth conditions, measuring technique and other environmental or measurement parameters may have a profound, but undetermined impact on the behavior of the cell and the resulting dataset. Consequently, these approaches often resort to annotation databases to determine the functionality of a module or TF. For example, in the GRAM method [5], where the functionality of a module is based on enrichment in MIPS functional categories, the TF Hap4 was only related to respiration. We could, on other hand, not only demonstrate that oxygen plays an important role, but also identified the known effect of the extracellular glucose concentration on Hap4 and its regulon.

In this study we identified many novel putative regulatory relationships. Examples include the role of Tye7 in regulating sulfur metabolism and the cross-regulation between phosphate and sulfate metabolism. Given the quality and uniqueness of the dataset, many other clues about regulation mechanisms related to yeast's metabolism and respiration can still be extracted.

We believe that quantification of the complex relationships that control cellular adaptation to different environments necessitates well-designed and carefully controlled experiments. In this respect, the design of experimental setups, where interrelated cultivation conditions are systematically combined, is especially important. The analysis of the individual and combined effects of the cultivation parameters in such experiments will help to reveal the multi-faceted nature of cellular regulatory mechanisms.

## Methods

### Data

#### Gene expression data

The employed microarray gene expression data consists of the measured transcriptional response of the yeast *Saccharomyces cerevisiae *to growth limitation by four different macronutrients (carbon, nitrogen, phosphorus and sulfur) in both the presence of oxygen (aerobic growth) and the absence of oxygen (anaerobic growth) [13]. The yeast is grown in chemostat cultures, which allow for the accurate control of the environmental parameters, i.e. concentrations of nutrients can be kept constant, as well as the pH value, the temperature and the growth rate. Three independently cultured replicates were performed per experimental condition. A complete description of the experimental procedures can be found in [12,13,30]. The systematic setup of the eight experiments is displayed in Figure 1. Sampling of the chemostat cultures, probe preparation and hybridization to Affymetrix GeneChip microarrays was performed as described previously [12]. Acquisition and quantification of array images and data filtering were performed using Affymetrix Microarray Suite Version 5.0. Before comparison, all arrays were globally scaled to a target value of 150 using the robust average signal from all gene features. The array data used in this study can be retrieved at Genome Expression Omnibus [31] with series numbers GSE4807 and GSE1723.

#### Transcription factor data

In [14] a combination of genome-wide location analysis (based on ChIP-chip technology) [4], motif discovery tools and literature was employed to recognize motifs in promoter regions that are bound by one of 102 known TFs. The resulting regulatory map indicates the number of motifs in the promoter region of a gene for a TF for all gene-TF pairs. We binarized this map such that an element indicates whether a gene can be bound by a TF or not. We employed only motifs that are bound with high confidence (*P *≤ 10^-3^); not taking into account conservation among other *sensu stricto Saccharomyces *species, since our interest in purely focused on *Saccharomyces cerevisiae*. The data was downloaded from [32].

#### Gene annotation data

Genes were associated with the processes in which they participate as annotated in Gene Ontology biological processes [10,33], MIPS functional categories [11,34] and KEGG pathways [35,36].

### Methodology

#### Selection of differentially expressed genes

Genes that show differential expression across the experimental conditions are selected. For this purpose, we employed a multi-class SAM analysis [37]. Here, the classes are the eight different experimental conditions. The 2500 most significantly changed genes are selected (median false discovery rate of 0.01%). This is an estimate of the number of genes involved in the metabolic processes of yeast grown under the experimental conditions [13].

#### Isolation of the global oxygen effect

To investigate the linear relationship between the aerobic and anaerobic expression values of a gene, we perform the following steps: First, we compute the mean and standard deviation across the replicates, *μ*_*ij *_and *σ*_*ij*_, for the nutrient limitations *i *= 1...4 and both aerobic (*j *= 1) and anaerobic (*j *= 2) growth. We model the joint aerobic-anaerobic expression distribution for each nutrient limitation *i *as a normal distribution *N*(*μ*_*i*_, ∑_*i*_), with *μ*_*i *_= [*μ*_*i*1_, *μ*_*i*2_] and ∑i=(σi1200σi22)
 MathType@MTEF@5@5@+=feaafiart1ev1aaatCvAUfKttLearuWrP9MDH5MBPbIqV92AaeXatLxBI9gBaebbnrfifHhDYfgasaacH8akY=wiFfYdH8Gipec8Eeeu0xXdbba9frFj0=OqFfea0dXdd9vqai=hGuQ8kuc9pgc9s8qqaq=dirpe0xb9q8qiLsFr0=vr0=vr0dc8meaabaqaciaacaGaaeqabaqabeGadaaakeaadaaeqaqaaiabg2da9maabmaabaqbaeqabiGaaaqaaGGaciab=n8aZnaaDaaaleaacqWGPbqAcqaIXaqmaeaacqaIYaGmaaaakeaacqaIWaamaeaacqaIWaamaeaacqWFdpWCdaqhaaWcbaGaemyAaKMaeGOmaidabaGaeGOmaidaaaaaaOGaayjkaiaawMcaaaWcbaGaemyAaKgabeqdcqGHris5aaaa@3ED7@. This is graphically depicted in Figure 2b. Next, we estimate the parameters of a linear model (slope and offset) by fitting a straight line through the four normal distributions. This heteroscedastic regression problem is solved as described in [38]. As a goodness-of-fit criterion for the regression, a P-value was computed by employing the Student's T cumulative distribution function with the ratio between the slope and the standard deviation of the slope. The P-value cut-off was set at 10^-4^. When no significant linear relationship (*P *> 10^-4^) is found employing the four nutrient limitations, we successively leave one of the nutrient limitations out, thus employing only three normal distributions to find a linear relationship. If *P *≤ 10^-4 ^for the best of the resulting four fits, this fit is used. This strategy handles genes with one nutrient-limitation-specific reaction to oxygen presence. See Additional file 7. When again no good linear relationship is found, the slope is fixed to one and only the offset (i.e. the difference between the mean aerobic and anaerobic expression level) is computed. See Additional file 8. The three different regression strategies (use of four nutrient limitations, use of three nutrient limitations, only compute the offset) were applied to 1190, 518 and 792 genes, respectively. For each gene, we apply the estimated parameters (slope *a *and offset *b*) to map the original aerobic expression values **x **to their linearly mapped values **x**', via **x**' = *a*·**x **+ *b*, thereby aligning the aerobic and anaerobic expression patterns, such that the differences in the resulting expression pattern are not caused by the oxygen effect. See for example, Figure 2c.

#### Construction of the discretized representation

A gene is represented by a ternary expression pattern of length nine. The first eight entries represent the discretized representation of the linearly mapped continuous expression data, which can be either 0, -1 or 1, indicating the most common expression level, downregulation or upregulation, respectively. Since the linear mapping changes the continuous expression pattern of a gene, SAM is run again on the linearly mapped data. Genes that now drop out of the top 2500 most differentially expressed genes are assigned a value of zero in the first eight entries of the expression pattern. Genes, that remain in the top 2500 (2062 genes) are discretized by employing k-means clustering for each gene separately, i.e. in an one-dimensional space on the eight mean expression levels associated with the eight experimental conditions. (Red crosses on the right vertical axis in Figure 2c). The Davies-Bouldin index [39] was used to choose between *k *= 2 (most common level and down- or upregulation) and *k *= 3 (all three quantized levels). Genes for which no compact and well-separated clusters could be found, i.e. for which the Davies-Bouldin index for both *k *= 2 and *k *= 3 exceeded 0.5, were removed. The most common level (0) was assigned to the experimental conditions that formed the largest cluster. The clusters with higher or lower gene expression levels with respect to the most common level cluster are labeled as upregulated (1) or downregulated (-1) respectively. The ninth entry of the discretized expression pattern of a gene represents the global oxygen effect. This can either be 0,-1 or 1. No significant difference between expression under aerobic and anaerobic growth is indicated by a zero (0). A consistent significantly lower or higher expression level when grown anaerobically is indicated by -1 and 1, respectively. The global oxygen effect is determined by performing pairwise T-tests for all nutrient limitations, comparing the original expression levels when grown aerobically with those when grown anaerobically. See Figure 2d. When at least three of the four nutrient limitations have a significantly (*P *≤ 5·10^-2^) higher expression when grown aerobically (or anaerobically) we assign a 1 (or -1 respectively). (In the case where only three nutrient limitations were used in regression only two of these three should be significantly higher (or lower) to pass the test.)

#### Generation of the modules

Modules are formed by grouping genes with identical discretized expression patterns, i.e. by performing a hierarchical clustering on the discretized data with Hamming distance as dissimilarity measure and then forming clusters by cutting the dendrogram at a distance of zero (linkage is irrelevant). Additionally, modules are formed with the global oxygen effect being irrelevant, i.e. genes are clustered together when only the first eight entries of the expression pattern are identical. Similarly, modules are created based solely on the oxygen effect. This strategy creates overlapping clusters of genes, that represent different characterizations based on the global oxygen effect.

#### Identification of significant TFs and enrichment of annotation categories

Modules are related to TFs by the hypergeometric test, which assesses the probability that the observed frequency that the genes in a module are bound by a TF would occur by chance. The P-value cutoff to decide whether a relation is significant is *P *≤ 1/(*n*_*m *_*n*_*x*_), where *n*_*m *_is the number of modules consisting of more than ten genes and *n*_*x *_is the number of TFs or TF pairs that bind to more than ten genes. This Bonferroni correction for multiple testing results in a per-family error rate (PFER) of one [40]. Considering the stringency of the Bonferroni correction and the fact that the tests are not independent, the P-value correction is quite conservative. The same procedure is employed to assess the overrepresentation of GO, MIPS and KEGG annotation categories.

#### Motif discovery

RSAT motif discovery [41] was applied to modules, which are significantly related to at least one TF or TF pair. An oligonucleotide analysis was run with motif sizes ranging from five to eight. Significant (RSAT occurrence significance score larger than one) and dissimilar motifs for each module were manually extracted. Published PWM/PSSM matrices for known TFs [14,42,43] are captured in the weight matrix form as described in [44]. A simple similarity score between a motif and a weight matrix, i.e. the sum of the weights of the matrix for the letters of the aligned motif, was employed to relate the uncovered motifs to known TFs.

## Authors' contributions

TAK devised the methodology and drafted the manuscript. JHW interpreted the results and assisted in structuring the manuscript. JMD carried out the microarray work and interpreted the results. PDL assisted in structuring the manuscript. JTP assisted in structuring the manuscript. MJTR participated in devising the methodology and helped to draft the manuscript. LFAW participated in devising the methodology and helped to draft the manuscript. All authors read and approved the final manuscript.

## Supplementary Material

Additional file 1 **Discretized Clustering With Linear Mapping**. An Excel file containing all the modules derived with the proposed method. Also, significantly enriched annotation categories and transcription factors are given. Click here for file

Additional file 6 **Microscopic pictures of Saccharomyces cerevisiae**. Microscopic pictures of *Saccharomyces cerevisiae *grown in aerobic carbon limited (left) and anaerobic carbon limited (right) chemostats. The cells were sampled from the fermenters and directly observed under an optical microscope equipped with a camera. Also for the other nutrient limitations these observations were made. These results were not photographed. Click here for file

Additional file 2 **Discretized Clustering Without Linear Mapping**. An Excel file containing all the modules derived with the proposed method, however without applying the linear mapping. Also, significantly enriched annotation categories and transcription factors are given. Click here for file

Additional file 4 **Enrichment of annotation categories with and without appliance of the linear mapping**. After performing the hypergeometric tests on modules created both with and without appliance of the linear mapping, we select for all different annotation categories from GO, KEGG and MIPS, the smallest P-value (highest enrichment in a particular module) for both approaches. These minimal P-values for all functional categories obtained by applying or omitting the linear mapping are plotted against each other. Note that in the case of the Gene Ontology (GO) we consider two types of data: One indicates whether a gene is assigned to a particular leaf (biological process) in the GO annotation tree. This one is referred to as 'GO leaves'. The other associates a gene located in a certain leaf not only with that particular leaf but also with all nodes between the leaf node and the root of the GO tree. We refer to this GO data as 'GO comp'. Click here for file

Additional file 5 **The TF circle for modules uncovered without applying the linear mapping**. Similar to Figure 4, except now the proposed method is applied without performing the linear mapping. Click here for file

Additional file 7 **Procedure to derive the discretized representation of a gene using only three nutrient limitations in the regression**. Similar to Figure 2. For this gene, no good linear relationship could be found using all four nutrients limitations. However, when the carbon limitation is left out, there exist a good linear relationship (see **b**). Click here for file

Additional file 8 **Procedure to derive the discretized representation of a gene using the mean-offset correction**. Similar to Figure 2. For this gene, no good linear relationship could be found using four or sets of three nutrient limitations. Therefore, the slope is fixed to one and only the offset is computed (see **b**). Click here for file

Additional file 3 **Permutation tests**. For the differentially expressed genes in the dataset, the estimated parameters (scaling factor (slope) and offset) from the heteroscedastic regression are compared with those generated by employing the regression strategy on 1000 datasets, where the eight condition labels were randomly permuted. Also, the P-values that represent the variability of the slope and the offset were computed for all permutations. In the top-left plot the red line indicates the number of genes with a P-value that is lower than the *P*_*cutoff*_, which is found on the x-axis. The blue line indicates the false discovery rate (FDR) as a function of the *P*_*cutoff*_. Here, the FDR is defined as the median number of genes with *P *<*P*_*cutoff *_from the permuted datasets divided by number of genes with *P *<*P*_*cutoff *_from the original dataset. The top-right plot displays the same features for the offset. In these top figures, if the scaling factor is smaller than zero, the P-values of scaling factor and offset are set to 1. A slope smaller than zero implies that the best linear relationship is found when inverting the expression pattern. This, of course, makes no sense from a biological perspective. The bottom-left plot displays the distribution of the computed scaling factors. This distribution is estimated using Parzen density estimation. From the 1000 permuted datasets, 1000 different distributions were estimated. These were plotted using an errorbar plot, which indicates the standard deviation of the permuted distribution. Here, it is clearly visible that for the correct labels only few genes exhibit a slope smaller than zero. Additionally, for most genes the slope is between zero and one and the offset is smaller than zero, indicating that the majority of genes have a higher expression when grown within the presence of oxygen. The bottom-right plot displays the original and permuted distribution for the offset. Click here for file

## Figures and Tables

**Table 1:** Overview of the uncovered modules

Module		Disc.Expr.Pattern									TF binding		Motif Discovery		Annotation		
		Aer				Ana											

no.	# genes	C	N	P	S	C	N	P	S	Ox	TFs	TFpairs	Motif	Ass.TFs	GO	MIPS	KEGG

1	57	1	0	0	0	1	0	0	0	1	Hap4		CCAATCA	Hap5, Hap2/3/4, Mcm1	GO: Oxidative phosphorylation		
													ATTGG	Hap5, Hap2/3/4, Mcm1, ...	MIPS: Respiration		
															KEGG: Oxidative phosphorylation		

2	70	0	1	0	0	0	1	0	0	0	Dal82	Gln3-Dal82	AGATAAG	Gzf3, Dal80, Gat1	GO: Catabolism		
											Gln3		CTTATC	Gat1, Gzf3, Dal82, ...	MIPS: Nitrogen and sulfur utilization		
															KEGG: Cyanoamino acid metabolism		

3	211	0	0	0	0	0	0	0	0	1	Hap1						

4	70	0	0	0	1	0	0	0	1	0	Cbf1	Met32-Cbf1	CACGTGA	Cbf1, Tye7, Ino4, ...	GO: Sulfur metabolism		
											Met32	Yap7-Yap1	GCCACA	Met4, Rpn4	MIPS: Amino acid metabolism		
											Yap7				KEGG: Sulfur metabolism		

5	44	0	0	1	0	0	0	1	0	0	Pho4	Pho4-Cbf1	ACGTGC	Pho4, Cbf1, Ino2, ...	GO: Anion transport		
													CACGTGG	Pho4, Tye7, Cbf1, ...			

6	15	0	0	0	1	0	0	0	1	1	Met32		GCCAC	Rpn4, Met4, R. car1, ...			
													CTGTGGC	Met4, Rfx1			

7	169	1	0	0	0	1	0	0	0	x	Hap4		GGGGTA	Mig1, Rap1	GO: Oxidative phosphorylation		
													ACCCC	Mig1, Adr1, Msn4, ...	MIPS: Respiration		
															KEGG: Oxidative phosphorylation		

8	100	0	1	0	0	0	1	0	0	x	Dal82	Gln3-Dal82	CTTATC	Gat1, Gzf3, Dal82, ...	GO: Amine transport		
											Gln3		AGATAAG	Gzf3, Dal80, Gat1	MIPS: Nitrogen and sulfur utilization		

9	93	0	0	0	1	0	0	0	1	x	Cad1	Met32-Cbf1	GCCACA	Met4, Rpn4	GO: Sulfur metabolism		
											Cbf1	Met32-Met31	CACGTGA	Cbf1, Tye7, Ino4, ...	MIPS: Metabolism of methionine		
											Met31	Tye7-Cbf1	CTGTGGC	Met4, Rfx1	KEGG: Sulfur metabolism		
											Met32						
											Met4						
											Yap7						

10	52	0	0	1	0	0	0	1	0	x	Cbf1	Pho4-Cbf1	ACGTGC	Pho4, Cbf1, Ino2, ...	GO: Anion transport		
											Pho4		CACGTGG	Pho4, Tye7, Cbf1, ...			

11	638	x	x	x	x	x	x	x	x	1	Hap1		CCGATA	Hap1	GO: Oxidative phosphorylation		
											Hap4				MIPS: Respiration		
															KEGG: Oxidative phosphorylation		

12	383	x	x	x	x	x	x	x	x	-1	Dig1	Cin5-Aft2	ACAATAG	Yox1, Rox1	GO: Lipid metabolism		
											Rox1	Rox1-Cin5	TGCTTT	Upc2	MIPS: Metabolism		
											Ste12	Swi4-Mcm1					
											Swi4	Tec1-Dig1					
											Tec1	Tec1-Ste12					
												Yap6-Cin5					

13	3883	0	0	0	0	0	0	0	0	0	Fhl1	Rap1-Fhl1	AAAAT	Rlr1, Spt23			
												Sfp1-Fhl1	GAAAA	Rlr1, Ume1, Azf1, ...			
													AAAAA	Azf1, Sig1, Met4			
													TGAAA	Ste12, Dig1, Ume1, ...			
													AAATA	Smp1, Rlm1, Azf1, ...			
													AAATT	Pho2, Spt23			

Detailed information for all modules that are significantly related to at least one TF(-pair). Besides the discretized expression pattern and the significant TFs from binding data, the table reports overrepresented motifs through motif discovery as well as TFs associated to these motifs. Also, the most highly enriched GO, MIPS and KEGG category for each module is given (if significant).

**Table 2:** Effect of the linear mapping on module size and enrichment

Cultivation parameter	Linear mapping applied												No linear mapping										
	Disc.Expr.Pattern									# genes	# TF(pairs)	# Ann.cat	Disc. Expr. Pattern								# genes	# TF(pairs)	# Ann.cat

Carbon	1	0	0	0	1	0	0	0	x	169	1	48	1	0	0	0	1	0	0	0	59	-	5
	-1	0	0	0	-1	0	0	0	x	69	-	-	-1	0	0	0	-1	0	0	0	23	-	-

Nitrogen	0	1	0	0	0	1	0	0	x	100	2(1)	8	0	1	0	0	0	1	0	0	42	2(1)	8
	0	-1	0	0	0	-1	0	0	x	2	-	-	0	-1	0	0	0	-1	0	0	0	-	-

Sulfur	0	0	1	0	0	0	1	0	x	52	2(1)	1	0	0	1	0	0	0	1	0	39	2(1)	6
	0	0	-1	0	0	0	-1	0	x	2	-	-	0	0	-1	0	0	0	-1	0	1	-	-

Phosphorus	0	0	0	1	0	0	0	1	x	93	6(3)	27	0	0	0	1	0	0	0	1	59	5(3)	27
	0	0	0	-1	0	0	0	-1	x	4	-	-	0	0	0	-1	0	0	0	-1	1	-	-

Oxygen	x	x	x	x	x	x	x	x	1	638	2	75	1	1	1	1	0	0	0	0	115	2	19
	x	x	x	x	x	x	x	x	-1	383	5(6)	13	0	0	0	0	1	1	1	1	76	-	5

For the modules that are most straightforwardly related to one of the the cultivation parameters (the four nutrient limitations and the oxygen availability) this table indicates the size of the respective module, the number of associated TFs, TF pairs and annotation categories; both with and without appliance of the linear mapping. (Note that when no linear mapping is applied the original continuous expression levels are discretized and no oxygen effect can be computed, resulting in a discretized expression pattern of length eight.)
