# Supplementary figures and images for: Exploiting combinatorial cultivation conditions to infer transcriptional regulation
Source: BMC Genomics. 2007 Jan 22;8:25. doi: 10.1186/1471-2164-8-25 (PMC1797021; doi:10.1186/1471-2164-8-25)

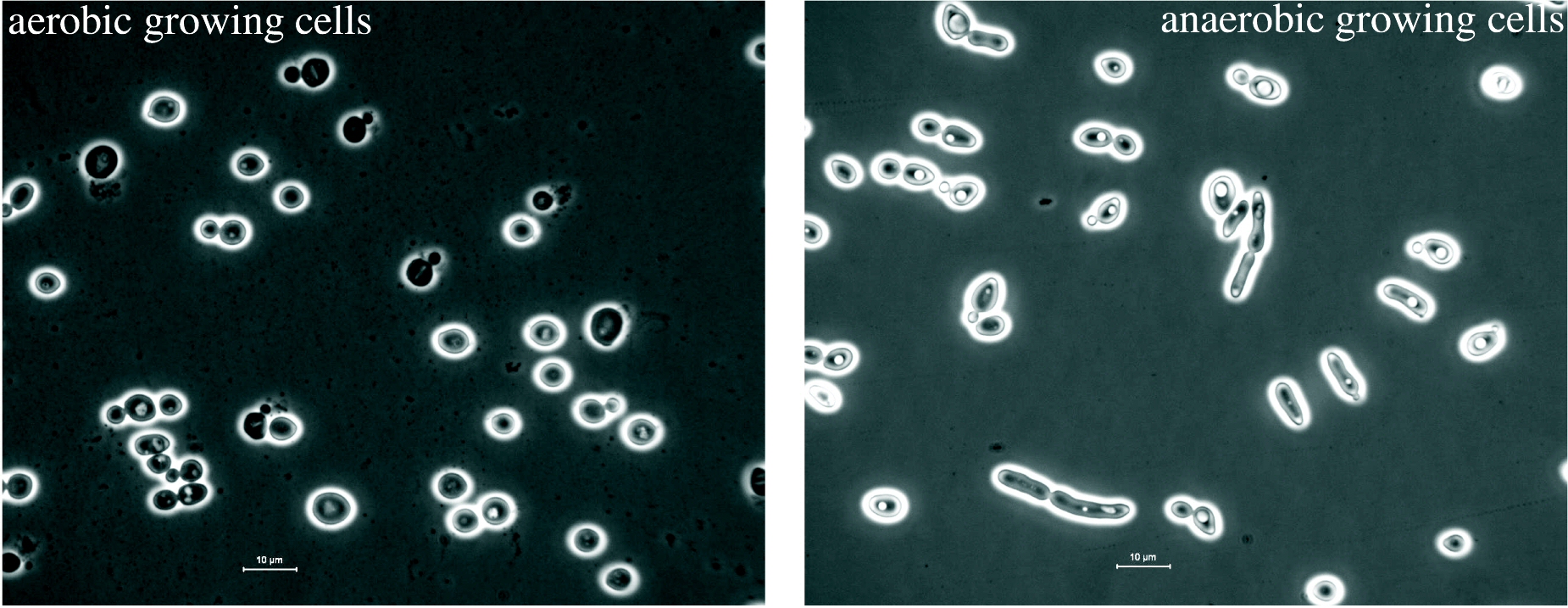

Supplement: Additional file 6 — Microscopic pictures of Saccharomyces cerevisiae. Microscopic pictures of Saccharomyces cerevisiae grown in aerobic carbon limited (left) and anaerobic carbon limited (right) chemostats. The cells were sampled from the fermenters and directly observed under an optical microscope equipped with a camera. Also for the other nutrient limitations these observations were made. These results were not photographed. [file 1471-2164-8-25-S6.jpeg]
